# Imaging flow cytometry reveals divergent mitochondrial phenotypes in mitochondrial disease patients

**DOI:** 10.1016/j.isci.2024.111496

**Published:** 2024-11-28

**Authors:** Irena.J.J. Muffels, Richard Rodenburg, Hanneke L.D. Willemen, Désirée van Haaften-Visser, Hans Waterham, Niels Eijkelkamp, Sabine A. Fuchs, Peter M. van Hasselt

**Affiliations:** 1 Department of Metabolic Diseases, Wilhelmina Children’s Hospital, University Medical Center Utrecht, Utrecht 3584 EA, the Netherlands; 2 Nijmegen Center for Mitochondrial Disorders, Radboud University Nijmegen Medical Center, Nijmegen 6525 GA, the Netherlands; 3 Center for Translational Immunology (CTI), Wilhelmina Children’s Hospital, University Medical Center Utrecht, Utrecht 3584 EA, the Netherlands; 4 Department of Pediatrics, Center for Lysosomal and Metabolic Diseases, Erasmus University Medical Center, Rotterdam 3015 GD, the Netherlands; 5 United for Metabolic Diseases (UMD), Utrecht 3584 EA, the Netherlands; 6 Department of Laboratory Medicine, Laboratory Genetic Metabolic Diseases, Amsterdam UMC - AMC, Amsterdam 1105 AZ, the Netherlands

**Keywords:** Health sciences, Medicine, Natural sciences, Biological sciences, Genetics, Human genetics

## Abstract

Traditional classification by clinical phenotype or oxidative phosphorylation (OXPHOS) complex deficiencies often fails to clarify complex genotype-phenotype correlations in mitochondrial disease. A multimodal functional assessment may better reveal underlying disease patterns. Using imaging flow cytometry (IFC), we evaluated mitochondrial fragmentation, swelling, membrane potential, reactive oxygen species (ROS) production, and mitochondrial mass in fibroblasts from 31 mitochondrial disease patients. Significant changes were observed in 97% of patients, forming two overarching groups with distinct responses to mitochondrial pathology. One group displayed low-to-normal membrane potential, indicating a hypometabolic state, while the other showed elevated membrane potential and swelling, suggesting a hypermetabolic state. Literature analysis linked these clusters to complex I stability defects (hypometabolic) and proton pumping activity (hypermetabolic). Thus, our IFC-based platform offers a novel approach to identify disease-specific patterns through functional responses, supporting improved diagnostic and therapeutic strategies.

## Highlights

• Imaging flow cytometry performs reliable, multimodal assessment of mitochondrial function
• 97% of 31 mitochondrial disease patients show aberrancies on mitochondrial assays
• Mitochondrial patient-derived fibroblasts show either a hypo- or hypermetabolic phenotype
• The hypermetabolic cluster showed significantly more neuropathy

## Graphical abstract

**Figure:**
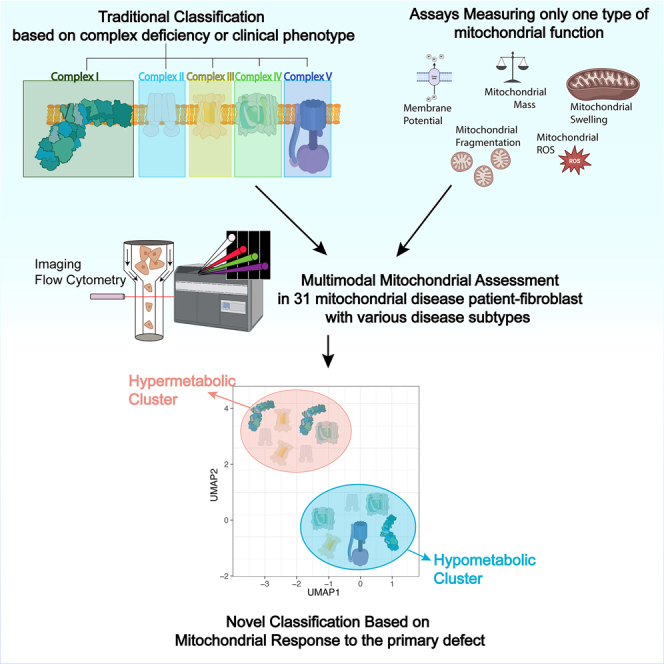

## Introduction

Mitochondrial diseases encompass a group of rare disorders characterized by dysfunctional energy metabolism, resulting from pathogenic genetic variants in nuclear or mitochondrial genes. For most genetic diseases, patients harboring the same disease subtype show similar phenotypes. Mitochondrial diseases appear to be an exception to this phenomenon, as patients with the same disease subtype or dysfunctional gene can present with completely different clinical and cellular phenotypes.^1^^,^^2^^,^^3^^,^^4^ Even more striking, patients harboring the exact same pathogenic variant can present with highly heterogeneous clinical features, best illustrated by patients harboring the prevalent m.3243A>G variant in *MT-TL1* (mitochondrial leucine tRNA), that can result in either isolated myopathy, diabetes, deafness or ataxia, but also in the syndromic combination of Mitochondrial Encephalomyopathy, Lactic Acidosis and Stroke-like episodes (MELAS).^5^ The substantial heterogeneity and complex genotype-phenotype correlations greatly complicate diagnosis, prognosis prediction and therapy development for mitochondrial diseases.

To understand genotype-phenotype correlations in mitochondrial disease, patients have been characterized according to their deficient oxidative phosphorylation (OXPHOS) complex. Nonetheless, heterogeneity persists among patients with similar complex deficiencies, illustrated by patients with isolated complex I deficiency that can have either increased or decreased membrane potential, mitochondrial number and/or mitochondrial fragmentation.^6^ Similar discrepancies were observed in fibroblasts of patients harboring complex V deficiency.^3^ This may relate to the fact that most OXPHOS complexes establish interactions with other complexes to allow supercomplex formation,^7^ to facilitate optimal electron transfer with limited Reactive Oxygen Species (ROS) formation.^8^ Supercomplex formation is especially important for complex I, as 80% of all complex I is usually present in supercomplexes, and its presence in supercomplexes significantly promotes complex I stability.^9^^,^^10^^,^^11^ Characterization based on complex deficiency is further complicated by the fact that complex specific subunits or assembly factors are generally not as specific as once thought. For example, NDUFA4, previously known as a complex I assembly factor, has also been identified as a complex IV associating factor.^12^ TMEM70, which was thought to be primarily a complex V assembly factor, is also involved in complex I assembly.^13^ The interdependency of OXPHOS complexes implies that complex-directed disease subtyping may not be ideal to understand the complex genotype-phenotype relationships in mitochondrial disease.

Mitochondria have many functions that go beyond OXPHOS complex mediated ATP production, which involve ROS production, mitochondrial morphology, lipid oxidation, lipid synthesis, redox homeostasis, Fe/S cluster synthesis, copper metabolism, cardiolipin metabolism, calcium uptake, and amino acid metabolism.^14^ Similar to the interdependency of OXPHOS complexes, all of these functional aspects are closely intertwined. Thus, understanding mitochondrial pathophysiology asks for a comprehensive approach, omitting the traditional characterization based on one single functionality, and taking into account the various aspects of mitochondrial function. While several approaches to comprehensively quantify mitochondrial health have been proposed^15^^,^^16^^,^^17^^,^^18^^,^^19^, these techniques have primarily focused on one or two features—three at most—and have not yet been tested in patients with primary mitochondrial disorders.

Here, we developed a multifaceted assessment of mitochondrial morphology and function using Imaging Flow Cytometry (IFC). IFC combines the high-throughput sampling of flow cytometry with image acquisition, and the easy-to-use data analysis program allows for rapid and straightforward analysis of these images. We selected five features to assess aberrancies in mitochondrial morphology and function, validated specificity of these features using molecular compounds, and applied these features to 31 different patient-derived fibroblast lines. We identified two disease subtype-overarching clusters, characterized by either a hyper- or hypometabolic response to pathogenic mutations. Additionally, the two clusters correlated with specific clinical phenotypes. These results demonstrate that mechanistic exploration of cellular responses to mitochondrial disruptions is essential for understanding distinct genotype-phenotype relationships in mitochondrial diseases. Additionally, these divergent defense mechanisms may require their own unique diagnostic and therapeutic strategies, paving the way for more targeted approach of mitochondrial diseases.

## Results

### Assay selection

To quantify mitochondrial function and morphology in a multifaceted way, we first selected dyes and features that would reflect a broad spectrum of functional, morphological, and molecular domains of mitochondrial functionality. Dyes and features were based on the five most important qualifiers of mitochondrial function as described by Monzel et al.^14^ (Table 1).

**Table 1:** Overview of imaging dyes and features

Mitochondrial function	Fluorescent dye	Feature	Positive control
Membrane potential	Tetramethylrhodamine, methyl ester, perchlorate (TMRM)	TMRM intensity (normalized to Carbonyl cyanide-p-trifluoromethoxyphenylhydrazone (FCCP))	Rotenone (membrane potential ↓)^20^
Mitochondrial mass	Nonyl acridine orange (NAO)	NAO intensity	Valproic acid (mitochondrial mass ↑)^21^ Ethidiumbromide (mitochondrial mass ↓)^22^
Mitochondrial swelling	Tetramethylrhodamine, methyl ester, perchlorate (TMRM)	Mitochondrial area	Ethidiumbromide (mitochondrial swelling ↓)^22^
Mitochondrial fission/fusion/fragmentation	Tetramethylrhodamine, methyl ester, perchlorate (TMRM)	Form factor: perimeter^2^/(4π×area)	Staurosporine (fragmentation ↑)^23^ MDIVI (mitochondrial fusion ↑)^24^
Reactive oxygen species (ROS)	MitoSox	Non-nuclear MitoSox intensity	Antimycin-A (ROS production ↑)^25^

Table 1: showing imaging flow cytometry (IFC) dyes and features. The ↑ sign indicates that the molecular compound selected as positive control heightens the feature value. The ↓ sign indicates that the molecular compound selected as positive control decreases the feature value.

### Impact of fibroblast confluency and passage number on mitochondrial function

First, we evaluated the effect of fibroblast confluency and passage number on IFC features. When confluency dropped below 30% or exceeded 90%, all assays showed significant aberrancies (IQR <25^th^ percentile or >75^th^ percentile) (Figure S1A). Similarly, high passage number (>15) affected membrane potential, form factor and ROS production significantly (IQR <25^th^ percentile or >75^th^ percentile) (Figure S1B). To minimize confluency- and passage-induced effects, we ensured that confluency was kept between 30% and 90%, and passage number differences between donors were kept at a minimum (preferably <5, but at least <15).

### Assay validation

**Figure 1:**
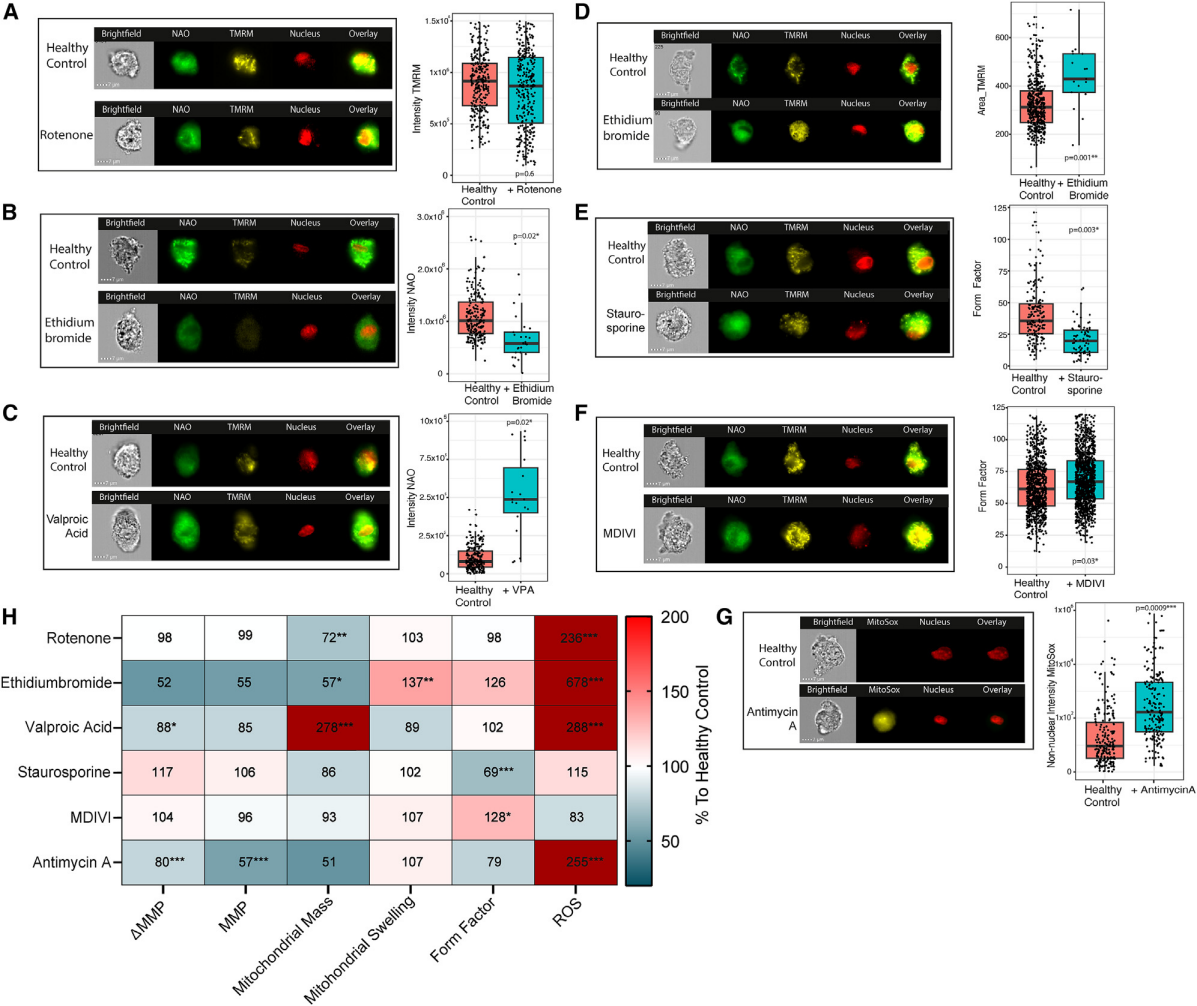
Assay validation using molecular compounds For all boxplots, the black line indicates the median value, the lower and upper hinges correspond to the 25th and 75th percentiles. The upper and lower whisker extend to 1.5∗IQR. Linear mixed model analysis was used to calculate significance (∗*p* < 0.05, ∗∗*p* < 0.01, ∗∗∗*p* < 0.001, *∗∗∗∗p* < 0.0001*).* (A–G) show a representative example of a healthy control fibroblasts and the same healthy donor treated with. (A) Rotenone for 4 h (10 ng/mL). The boxplot shows the non-normalized TMRM intensity for both conditions. (B) Ethidiumbromide for 7 days (25 ng/mL). The boxplot shows the NAO intensity in the FCCP treated condition for both conditions. (C) Valproic acid for 4 days (10 mM). The boxplots show the NAO intensity in the FCCP treated condition for both conditions. (D) Ethidiumbromide for 7 days (25 ng/mL). The boxplot shows the non-normalized TMRM area for both donors. (E) Staurosporine for 2 h (1.2 μM). The boxplot shows the form factor (mean perimeter^2^)/(4π × mean area) for both conditions. (F) MDIVI for 16 h (50 μM). The boxplot shows the form factor for both conditions. (G) Antimycin A for 5 min (10 μM) causing mitochondrial reactive oxygen species production. The boxplot shows the Intensity of the MitoSox staining for both conditions. (H) HeatMap showing experimental results for healthy controls treated with molecular compounds on all axes. Similar treatment conditions and features were used as stated in Figures 1A–1G to create the graph. The mean value of the compound-treated fibroblast donor was normalized to the healthy control analyzed within the same experiment and converted to percentages. Percentages corresponding to the color coding are shown on the right. The black squares indicate the primary feature for which the compound was selected. Membrane potential was quantified by subtracting the background TMRM intensity in a sample treated with FCCP from that in a non-FCCP treated sample (ΔMMP). The absolute membrane potential without subtraction of the FCCP sample is indicated as MMP. Linear mixed model analysis was used to calculate significance (∗*p* < 0.05, ∗∗*p* < 0.01, ∗∗∗*p* < 0.001, *∗∗∗∗p <* 0.0001).

For validation of IFC dyes and features, we used six different compounds each known to affect one of the five selected features (Table 1). All compounds, except for rotenone, affected the corresponding feature as expected, indicating that IFC features reflected their corresponding mitochondrial function (Figures 1A–1G). For rotenone, a similar membrane potential was observed after treatment in the non-apoptotic cellular population. (Figure 1A). In literature, highly variable effects of rotenone have been observed; however, most of these experiments did not take apoptosis into account, which might explain the different results compared to our study (Barrientos and Moraes, 1999). Next, we evaluated the effect of these compounds on other features than the ones for which they were primarily selected. We found that all compounds—including rotenone—affected multiple features (Figure 1H), supporting the notion that mitochondrial functions are mutually dependent.

### Characteristics of the patient cohort

**Figure 2:**
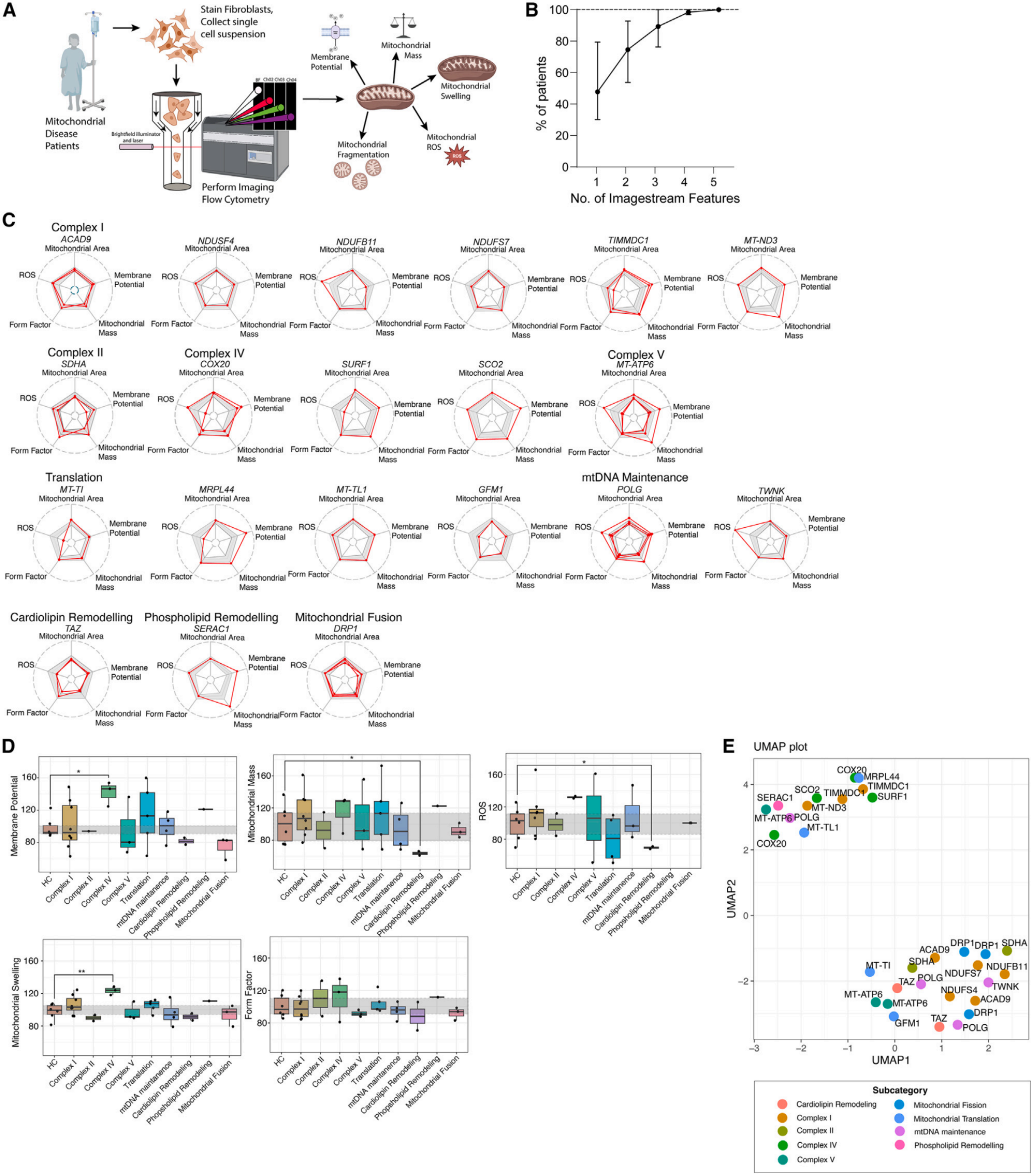
Results of imaging flow cytometry (IFC) assays for patients with mitochondrial disease (A) Graphical representation of the IFC workflow. Fibroblasts derived from mitochondrial disease patients were stained with NAO, TMRM, MitoSox, and DRAQ5. Living cells were analyzed using IFC and the five different mitochondrial features were extracted from the software. (B) The percentage of patients showing significant changes on one or more assays was assessed for single features and for feature combinations. Changes were considered significant when the observed values fell outside the range of healthy controls. The y axis shows the percentage of patients with significant changes on either one, two, three, four, or all five IFC assays. The dots represent the mean percentage for all possible combinations, the error bar represents the range. (C) Radar plots showing the results for each patient on the five IFC assays. To create the radar plots, the values of each of the five features (Table 1) were normalized against the mean of the healthy control taken along within the same experiment and converted to percentages. Patients harboring genetic variants in the same gene are grouped together. The light gray planes indicate the range of aberrancies in healthy control (lowest-highest). The line in the middle of the gray plane indicates the mean values in healthy controls. The red lines refer to the patient values. (D) Boxplots showing IFC assay results for all five assays. For the boxplots, the values of each of the five features (Table 1) were normalized against the mean of the healthy control taken along within the same experiment and converted to percentages. The y axis refers to the percentage observed in that specific donor normalized against the healthy control taken along in the same run. All six healthy controls (HC) were normalized against one healthy control. Each dot represents one donor. The gray planes indicate the reference values, based upon the IQR25-IQR75 values of six healthy controls. The black line indicates the median value, the lower and upper hinges correspond to the 25th and 75th percentiles. The upper and lower whisker extend to 1.5∗IQR. Statistics were only calculated when disease subgroups consisted of three patients or more. Mann-Whitney U test was used to calculate significance. Each group was compared to the healthy control reference range. Only significant values are marked. (E) UMAP (Euclidean distance, neighbors = 8, minimal distance = 0.01) showing IFC results for all patients with mitochondrial disease. As input, the normalized percentage values were used (Table S1 column C–G). The dots are colored according to the disease subtypes. The text refers to the name of the mutated gene.

**Figure 3:**
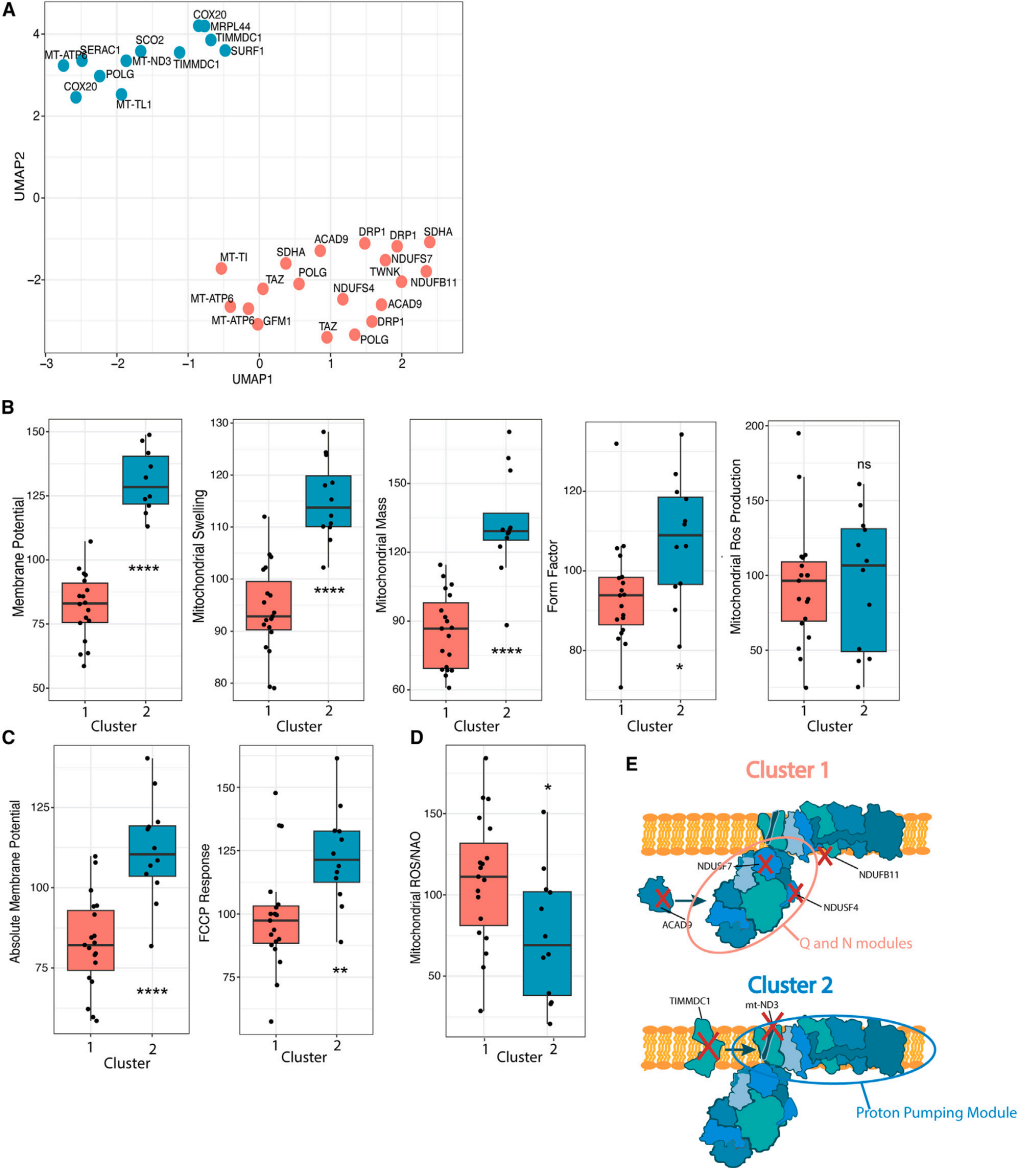
Clustering of mitochondrial disease patients (A) Showing the same UMAP as in Figure 2E; however, now the nodes are colored according to the clusters. Distance was calculated using Euclidean distance, and Ward’s method was used to identify clusters. (B) Boxplots showing the IFC characteristics for each of the two clusters. Statistics were calculated using Welsch’s t test. The black line indicates the median value, the lower and upper hinges correspond to the 25th and 75th percentiles. The upper and lower whisker extend to 1.5∗IQR. (C) Boxplot showing the FCCP response for each of the two clusters. For the boxplots, the values of each of the five features (Table 1) were normalized against the mean of the healthy control taken along within the same experiment and converted to percentages. The FCCP response was calculated by dividing the mean TMRM intensity of the untreated sample by the mean TMRM intensity of the FCCP treated sample. This ratio was then compared to the healthy control taken along in the same run and converted to percentage values. (D) Boxplot showing mitochondrial ROS production normalized against mitochondrial mass for the two clusters. For the boxplots, the values of each of the five features (Table 1) were normalized against the mean of the healthy control taken along within the same experiment and converted to percentages. Since MitoSox and TMRM were analyzed in two separate experiments, intensity values were first normalized against healthy controls, and these normalized values were then converted to ratios by dividing the normalized MitoSox values by the normalized NAO intensity values. This ratio is shown on the Y axis. The black line indicates the median value, the lower and upper hinges correspond to the 25th and 75th percentiles. The upper and lower whisker extend to 1.5∗IQR. (E) Graphical representation of complex I and the location of the pathogenic variants of patients harboring isolated complex I deficiency (indicated with a red cross). In cluster 1, patients with mutations affecting the N or Q module are included, leading to defective complex I assembly and stability (red circle). In cluster 2, patients with mutations affecting the P module are included, affecting proton pumping activity (blue circle).

**Figure 4:**
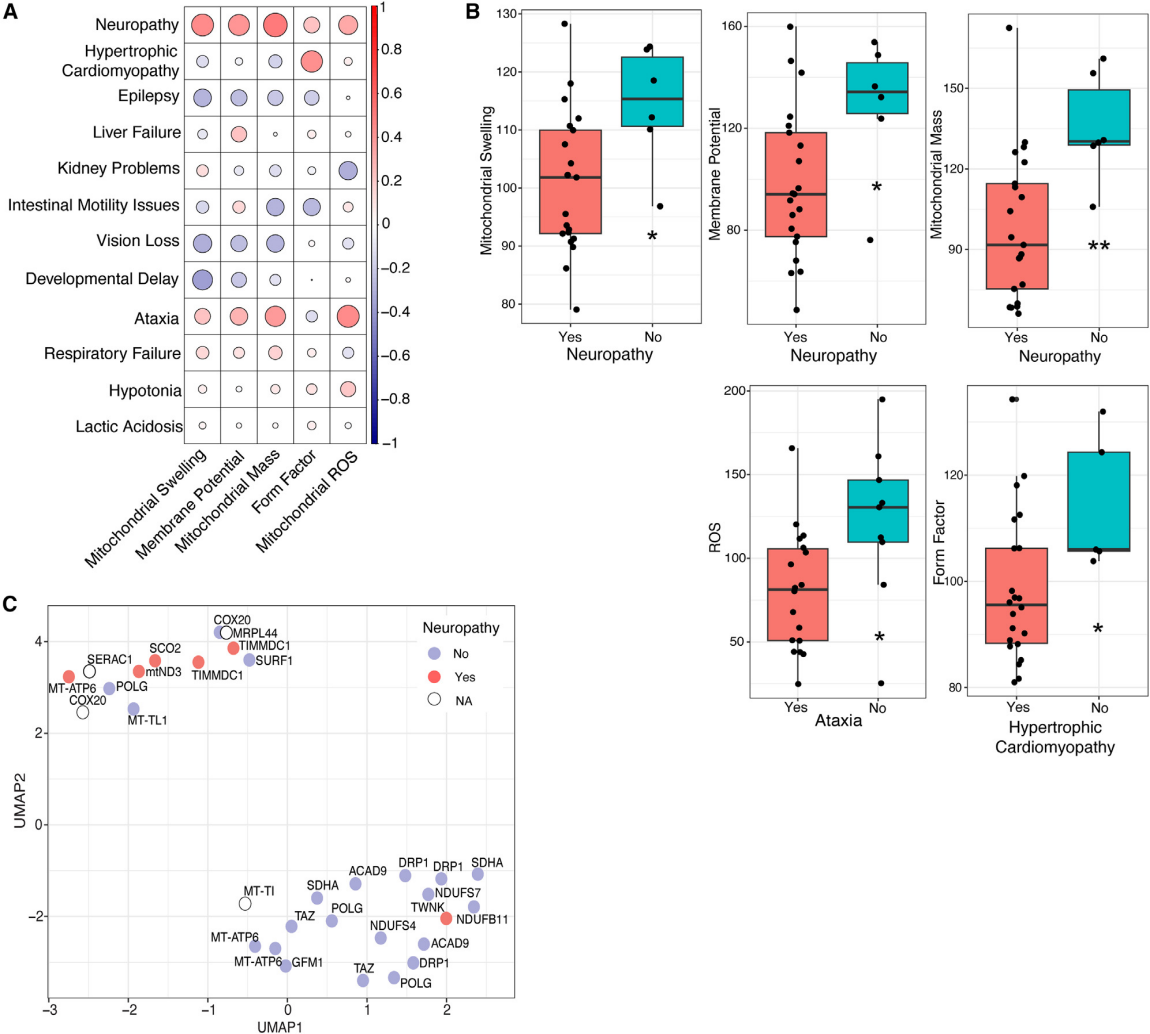
Correlation of IFC features with phenotypic features (A) Correlation plot showing the correlation between IFC features and clinical phenotypes. All clinical features were converted to binary parameters (Yes = Present, No=Not present) and correlation was calculated using Spearman’s correlation. The color coding and dot size correlate with Spearman’s rho coefficient. (B) All significant correlations have been plotted as a boxplot. For the boxplots, the values of each of the five features (Table 1) were normalized against the mean of the healthy control taken along within the same experiment and converted to percentages. Statistics were calculated using Welsch’s t test. The black line indicates the median value, the lower and upper hinges correspond to the 25th and 75th percentiles. The upper and lower whisker extend to 1.5∗IQR. (C) Showing the UMAP plots from Figures 2C and 3A. The nodes are colored according to the presence of neuropathy (Yes, No, NA). NA means that the specific feature was not assessed in patients. Statistical significance between the two clusters was calculated using Fisher’s exact test. Neuropathy was the only clinical feature that had a significant association with one of the clusters.

We included fibroblasts of 31 patients encompassing a range of different disease subtypes including isolated complex deficiencies (complexes I, II, III, IV, and V), as well defects of mitochondrial protein translation, mtDNA maintenance, and cardiolipin remodeling (Figure 2A). Clinical characteristics of the cohort are detailed in Table S1. Additionally, we included six healthy control fibroblast lines: four pediatric donors and two adult donors aged 20 and 23 years. The values found in the controls were normalized against the individual means and converted to percentages, and the lowest and highest percentage values were used as upper and lower reference values (Table S1). For mitochondrial disease patients, feature values of each of the five features (Table 1) were normalized against the mean of the healthy control taken along within the same experiment and converted to percentages. For Figures 2, 3, and 4, all values shown refer to these percentages, which are also shown in Table S1. Values were considered abnormal when they exceeded the lower or upper reference range established in six healthy controls.

### Imaging flow cytometry in mitochondrial disease patients

In patient-derived fibroblasts, we found that all five features could be either increased or decreased compared to healthy control fibroblasts (Table S1). When using one feature in isolation, 20%–60% of patients showed significant differences compared to healthy controls, depending on the feature used (Figure 2B). When considering all five features, 97% of patients showed significant changes on at least one of the five assays (Figure 2B). Table 2 shows the features that were most commonly aberrant in different mitochondrial disease subtypes compared to healthy controls. A subset of features had significant correlations with each other within the patient cohort, although rho values never exceeded 0.7 (Figure S2).

**Table 2:** Overview of highest ranked features that allow separation of mitochondrial disease subtypes from controls

Disease Subtype	Highest ranked feature	Aberrant in % of patients	Highest ranked feature	Aberrant in % of patients	Highest ranked feature	Aberrant in % of patients
Complex II	Membrane potential	50%	Mitochondrial mass	50%	Form factor	50%
Mitochondrial translation	Membrane potential	60%	Mitochondrial swelling	60%	ROS	60%
Cardiolipin remodeling	Membrane potential	100%	Mitochondrial mass	100%	–	–
Complex IV	Mitochondrial swelling	100%	ROS	100%	–	–
Complex V	Membrane potential	100%	ROS	100%	–	–
mtDNA maintenance	Mitochondrial mass	75%	–	–	–	–
Complex I	Membrane potential	88%	–	–	–	–
Mitochondrial fission	Membrane potential	100%	–	–	–	–

Table 2: showing the highest ranked features that allowed separation of the mitochondrial disease subgroup from healthy controls. For some disease subgroups, multiple features were equally important in setting them apart from healthy controls, and multiple features are shown.

We then assessed the correlation of IFC features with traditional diagnostic testing for mitochondrial disease. In 19% of patients (6/31), seahorse experiments were performed as part of their diagnostic follow-up, although in none of these patients, significant differences in basal or maximal respiration (oxygen consumption rate [OCR]) were found (Table S1). In contrast, 5/6 patients showed significant changes on at least one of six IFC features (Figure S2B). We found a significant correlation between ROS production and isolated OXPHOS complex IV deficiency in muscle (Figure S2C).

### Correlation of IFC characteristics with disease subtypes

First, we assessed whether IFC aberrancies correlated with specific genetic defects or disease subtypes. Figure 2C shows an overview of the significant changes for each of the five features for all 31 patients, grouped per gene. The majority of patients with pathogenic variants in the same gene showed similar changes. We found that a small subset of disease subtypes showed similar changes on single axes (Figure 2D). Patients with complex IV deficiency showed increased membrane potential, while patients with *TAZ* variants, affecting cardiolipin remodeling, showed decreased mitochondrial mass and ROS production. We calculated similarity between patients with Euclidean distance, and performed dimensionality reduction using Uniform Manifold Approximation and Projection (UMAP), to see which patients would have a similar phenotype and would thus cluster together on UMAP. We found that most disease subtypes did not cluster together on UMAP, highlighting the divergent effects of different mitochondrial disease subtypes on mitochondrial function. (Figure 2E).

Next, we sought to identify disease subtype-overarching clusters showing similar changes on IFC assays. Hierarchical clustering revealed that mitochondrial disease patients could be divided into two clusters (Figure 3A). Cluster 1 was characterized by decreased membrane potential, while cluster 2 was characterized by increased membrane potential, increased mitochondrial swelling and increased mitochondrial mass (Figure 3B). The first cluster consisted of complex I, II, or V deficiencies, transcriptional deficits, and mutations in MT-TI, *DRP1*, or *TAZ* genes. The second cluster consisted of all patients harboring isolated complex IV deficiency, three patients with isolated complex I deficiency (*TIMMDC1* and *mt-ND3)*, one patient with complex V deficiency, one MELAS patient, and one patient with DNA polymerase subunit gamma (POLG) deficiency. Three patients in whom multiple complexes were affected were also part of cluster 2.^26^^,^^27^ While background-subtracted tetramethylrhodamine, methyl ester, perchlorate (TMRM) values differed significantly between clusters, the non-background subtracted membrane potential appeared similar (Figure 3C). The heightened membrane potential may therefore be associated with an enhanced response to FCCP rather than an absolute disparity in membrane potential (Figure 3C). Notably, heightened sensitivity to FCCP has been previously documented in patients harboring complex IV mutations.^28^ The administration of FCCP, known for inducing a substantial H+ current and abolishing the reversed action of complex V, provides an opportunity to unveil patients who rely on this compensatory mechanism to uphold membrane potential. Notably, all patients within cluster 1 exhibited an FCCP response similar to that of healthy controls, suggesting the absence of this compensatory mechanism. In support, the absence of reversed complex V activity has been observed in *NDUFS4* knockout mice.^29^ In contrast, all patients included in cluster 2 showed increased FCCP response, correlating with data obtained using SURF1-deficient fibroblasts.^28^ Together, these results indicate that the presence or absence of compensatory action of complex V could correlate with heightened membrane potential and increased FCCP response, contributing to the functional separation of the two clusters.

Patients with isolated complex I deficiencies were clustered separately, concurring with literature.^30^^,^^31^ The disparate phenotypes are thought to correlate with the location of the affected subunits within holo-complex I.^32^ Mutations leading to a disconnected N module cause increased ROS production, while mutations in the proton pumping module lead to decreased proton pumping activity and NADH accumulation. To assess whether these findings aligned with our clusters, we verified the location of the pathogenic variants within holo-complex I. Since cluster 1 was associated with increased ROS production over mitochondrial mass (Figure 3D), we hypothesized that this cluster would show complex I assembly defects and disconnection of the N module. Indeed, for patients with pathogenic *NDUFS4* and *NDUFS7* variants, disconnection of the N module has been observed.^32^^,^^33^ Similarly, pathogenic *NDUFB11* variants are associated with impaired assembly of the peripheral subunits, (including the N module) corresponding with the presence of this patient in cluster 1 (Figure 3E).^34^^,^^35^ Finally, pathogenic variants in *ACAD9* have been associated with accumulation of late-stage complex I assembly intermediates lacking the N module, explaining their presence in the first cluster (Figure 3E).^36^^,^^37^

In contrast, pathogenic *ND3* variants, part of cluster 2, generally affect proton translocation without accumulation of late-stage complex I assembly intermediates.^38^ Patients harboring pathogenic variants in *TIMMDC1* (part of cluster 2) are similarly thought to have dysfunctional proton pumping activity due to TIMMDC1’s involvement in the membrane arm of complex I (ND1) and lack of colocalization with late-stage intermediates.^39^^,^^40^

In conclusion, the separation of complex I deficient patients into two separate clusters could be related to functional defects caused by either disconnected N module or defective proton pumping activity.

### Correlation of mitochondrial phenotype with clinical phenotype

Next, we assessed whether IFC features correlated with specific clinical phenotypes (Figure 4A). We found five significant correlations (Figure 4B). Intriguingly, all except one patient (harboring a *TWNK* mutation*)* showing neuropathy were part of cluster 2 (Figure 4C, Fisher’s Exact test, *p* = 0.0027∗∗). For a subset of patients that were part of cluster 2, neuropathy was not assessed (NA) due to lethality at a very young age.

## Discussion

It has long remained challenging to capture mitochondrial disease pathophysiology by focusing on similar genetic mutations or OXPHOS complex deficiencies. We hypothesized that multifaceted functional screening of mitochondria in different disease subtypes could provide novel insights in the complex genotype-phenotype correlation of mitochondrial diseases. To this aim, we developed five IFC assays which we validated using molecular compounds known to disrupt one of these five features. We found that 97% of patients with mitochondrial disease showed significant changes on one or more of these five assays. While we did not observe disease subtype-specific phenotypes, we did identify two disease-overarching clusters, based on decreased membrane potential (cluster 1) or increased membrane potential, mitochondrial mass and mitochondrial swelling (cluster 2). Literature-supported analyses revealed that all genes in cluster 1 were associated with decreased complex I stability, whereas cluster 2 consisted of patients associated with decreased proton pumping activity. At the clinical level, we found that neuropathy was predominantly observed in cluster 2.

In conclusion, we here found that multifaceted quantification of mitochondrial features using IFC allows detection of specific functional defects in nearly all patients with mitochondrial disease. Additionally, the identification of disease subtype-overarching clusters based on similar responses to mitochondrial injury grants relevant insights into pathology, suggesting that functional characterization of mitochondrial diseases might be superior compared to genetic or complex based characterization.

The past decade, several attempts to perform high-throughput quantification of multiple aspects of mitochondrial morphology and function in patients with mitochondrial disease using live-cell imaging, microscopic screening or flow cytometry, have been made. (Bennett et al., 2023^41^; Iannetti et al., 2016; Koopman et al., 2005; Leipnitz et al., 2018; and Zuba-Surma et al., 2007^42^). Until now, most studies that use microscopy have only been applied to small patient groups, focusing on one subtype of disease, for example isolated complex I deficiency,^30^^,^^31^^,^^32^^,^^43^^,^^44^ *MFN2* mutations,^45^ or isolated complex V deficiency.^3^^,^^46^ Studies that did focus on multiple disease subtypes have used small patient groups only, which limits the reliable detection of disease-overarching clusters.^47^^,^^48^ While modern microscopic approaches could render equally effective results compared to IFC, the fact that this method has not been published makes it challenging to directly compare these approaches and its efficiency for detecting mitochondrial pathology in different disease subtypes. However, one large advantage could lie in the ability of newer IFC models to sort cells after performing mitochondrial analyses. With sorting, the mitochondrial DNA of specific cellular populations with similar mitochondrial functionality could be studied. Additionally, combining sorting with CRISPR screens could be used to identify novel variants or genes involved in mitochondrial function.^49^ Finally, IFC could be used to train classification models that can be used for so-called ghost cytometry, which makes use of machine learning approaches dependent on image-trained information to sort cells with conventional flow cytometry.^50^^,^^51^ The large advantage of using ghost cytometry over IFC with TMRM/nonyl acridine orange (NAO) would be that cells do not need to be stained and survive after sorting.

We used fibroblasts for our IFC assays. Although easily obtained and expanded, their glycolytic dependency might fail to unveil relatively mild mitochondrial disorders. However, our study shows that 97% of patients present with aberrancies on at least one of five assays, indicating that even in fibroblasts, mitochondrial aberrancies are recapitulated to some degree. The two clusters identified in this study reflect two convergent functional responses to mitochondrial pathology, which might best be characterized as either a hypo- or hypermetabolic response. While a hypermetabolic response to mitochondrial injury may seem controversial, it has been frequently observed in mitochondrial disease animal models^52^^,^^53^ and patient-derived cells.^54^^,^^55^ While the exact reason for this hypermetabolic state is not entirely clear, it is considered potentially damaging for mitochondrial disease patient cells, that struggle to maintain sufficient energy levels in the first place.^54^ Therefore, direct targeting of this potential harmful compensatory mechanism by decreasing hypermetabolism could be beneficial for patients. However, patients exhibiting a primarily hypometabolic state should not be exposed to these kinds of treatments. Instead, since cluster 1 was characterized by increased ROS production, these patients might benefit primarily from anti-oxidant treatment. Thus, our functional-based screening of mitochondrial disease patient cells could aid the optimization of personalized treatment strategies, aiming to normalize potentially harmful secondary deficits associated with primary pathology.

In conclusion, we here provide a high-throughput approach to quantify mitochondrial function in patients with mitochondrial disease using IFC. This approach enabled identification of abnormal mitochondrial features in nearly all patients with mitochondrial disease, and clustered patients based on similar functional responses to primary mitochondrial pathology. Our results indicate that functional classification of mitochondrial diseases may outperform genetic-based classifications, and can provide a novel basis to improve personalized diagnostic and therapeutic approaches for mitochondrial disease patients, based on shared secondary responses to mitochondrial injury.

### Limitations of the study

A limitation of our IFC approach is that only two donors per experiment were analyzed. To enable comparison across experiments, each result was normalized, allowing each patient to be compared to six healthy controls. However, comparing only two donors per experiment could introduce considerable noise, though 97% of patients surpassed these noise thresholds, indicating that significant abnormalities were still detectable. Milder phenotypes, however, may have been masked by this noise. Additionally, we analyzed patient-derived fibroblasts, which may not fully capture mitochondrial disease manifestations, ideally requiring disease-relevant tissues. Further, fibroblasts can exhibit varying heteroplasmy levels that fluctuate with culturing and aging, potentially affecting measurements. While we used cells with similar passage numbers to mitigate this, patient-derived cells may age faster, which could limit this adjustment. iPSC-derived cells, with stable heteroplasmy levels and reduced aging effects, could serve as a promising alternative for future studies.

## Resource availability

### Lead contact

Further information and requests for resources and reagents should be directed to and will be fulfilled by the lead contact, Peter M. van Hasselt (p.vanhasselt@umcutrecht.nl)

### Materials availability

This study did not generate new unique reagents.

### Data availability

• All normalized IFC results, clinical and biochemical data are included as Table S1. All raw IFC files (RIFs) and compensated IFC files (CIFs) can be found in: Figshare: https://doi.org/10.6084/m9.figshare.27058645.v1, Figshare: https://doi.org/10.6084/m9.figshare.27058630.v1, and Figshare: https://doi.org/10.6084/m9.figshare.27108961.v1.
• This paper does not report original code.
• Any other information required to reanalyze the data reported in this paper is available from the lead contact upon request.

## Acknowledgments

This work was funded by the Wilhelmina Children’s Hospital Stimulus Stipend 2019-2020 and the United For Metabolic Diseases Catalyst grant.

## Author contributions

Conceptualization: I.J.J.M., S.A.F., and P.M.v.H.; methodology: I.J.J.M., H.L.D.W., N.E., S.A.F., and P.M.v.H.; investigation: I.J.J.M., R.R., D.V.H.-V., and H.W.; visualization: I.J.J.M.; supervision: H.L.D.W., N.E., S.A.F., and P.M.v.H.; writing – original draft: I.J.J.M. and P.M.v.H.; writing – review and editing: I.J.J.M., R.R., H.L.D.W., D.V.H.-V., H.W., N.E., S.A.F., and P.M.v.H.

## Declaration of interests

The authors declare no competing interests.

## STAR★Methods

### Key resources table

**Table:** 

REAGENT or RESOURCE	SOURCE	IDENTIFIER
**Antibodies and dyes**		

TMRM	Sigma Aldrich	Cat#: T5428
NAO	Enzo Life Sciences	Cat#: ENZ-52306
MitoSox	Thermofisher	Cat#: M36008
DRAQ5	Biolegend	Cat#: 424101

**Biological samples**		

Patient derived fibroblasts and healthy control fibroblasts	Wilhelmina Children’s Hospital	

**Chemicals, peptides, and recombinant proteins**		

FBS	Sigma	Cat #F7524
FCCP	Targetmol	Cat#: T6834
Antimycin A	Sigma Aldrich	Cat#: A8674
Valproic Acid	Santa Cruz	Cat#: 1069-66-5
Ethidiubromide	Sigma Aldrich	Cat#: E1510
MDIVI	Sigma Aldrich	Cat#: M0199-5mg
Dialyzed FBS	Gibco	Cat#: 26400044
DMEM/F-12	Gibco	Cat #11765054
Penicillin-Streptomycin	Gibco	Cat#: 15140122
Trypsin	Gibco	Cat#: 15400054
TrypLE	Gibco	Cat#”: 12604021
M199 (for seahorse)	Gibco	Cat#: 11150067
Fetal Bovine Serum (for seahorse)	Gibco	Cat#: A5256701
Agilent Seahorse XF Base Medium	Agilent	Cat#: 103335-100
Glucose	Sigma Aldrich	Cat#: G7021
Sodium Pyruvate	Gibco	Cat#: 11360070
Glutamine	Life Sciences	Cat#: J60573.A1
Oligomycin	Sigma Aldrich	Cat#: O8376
FCCP	Sigma Aldrich	Cat#: 370-86-5
Rotenone	Sigma Aldrich	Cat#: 83-79-4
Antimycin A	Sigma Aldrich	Cat#: 1397-94-0

**Critical commercial assays**		

Bicinchoninic assay kit	Uptima	

**Software and algorithms**		

R 4.4.1	N/A	https://www.rstudio.com
ImageStream®X Mark II Imaging Flow Cytometer	Millipore	
Tecan Spark spectrophotometer	Tecan	
UVmc2 spectrophotometer	SAFAS	
RStudio 2024-04-2	N/A	https://www.rstudio.com
GraphPad Prism 10	N/A	https://www.graphpad.com/
ImageJ	N/A	https://imagej.net/ij/
IDEAS software Version 6.2	N/A	https://www.emdmillipore.com/US/en/20150122_174404
Adobe Illustrator	N/A	www.adobe.com
Normalized Imaging Flow Cytometry results, clinical and biochemical data of patients included in this study.	N/A	Table S1 of this paper
Raw and compensated Imaging Flow Cytometry dataset for NAO/TMRM experiments	IDEAS Version 6.2	Figshare: https://doi.org/10.6084/m9.figshare.27058645.v1
Raw Imaging Flow Cytometry dataset for MitoSox experiments	IDEAS Version 6.2	Figshare: https://doi.org/10.6084/m9.figshare.27058630.v1
Raw and compensated Imaging Flow Cytometry dataset for NAO/TMRM and MitoSox experiments for Molecular Compound Grid	IDEAS Version 6.2	Figshare: https://doi.org/10.6084/m9.figshare.27108961.v1

### Experimental model and study participant details

#### Human participants

Age and gender of individuals included in this study are shown in Table S1. There was no significant correlation (Spearman’s method) between age of the entire cohort and mitochondrial parameters (mitochondrial swelling *p* = 0.12, membrane potential *p* = 0.77, mitochondrial mass *p* = 0.09, form factor *p* = 0.14, ROS *p* = 0.4). There was also no significant correlation between age of healthy controls and mitochondrial features (mitochondrial swelling *p* = 0.2, membrane potential *p* = 0.43, mitochondrial mass *p* = 0.97, form factor *p* = 0.99, ROS *p* = 0.24). The correlations between sex and mitochondrial parameters for the entire cohort were not significant (mitochondrial swelling *p* = 0.14, membrane potential *p* = 0.7, mitochondrial mass *p* = 0.9, form factor *p* = 0.4, ROS *p* = 0.6). There were also no significant correlations between sex and mitochondrial parameters for the healthy control cohort (mitochondrial swelling *p* = 0.7, membrane potential *p* = 0.48, mitochondrial mass *p* = 0.27, form factor *p* = 0.86, ROS *p* = 0.9). Written informed consent was obtained from the parents of each child. Healthy fibroblast lines were recruited through the Wilhelmina Children’s Hospital metabolic biobank (TCBio 19–489/B, https://tcbio.umcutrecht.nl). All procedures performed in studies involving human participants were in accordance with the ethical standards of the institutional and/or national research committee(s) and with the Helsinki Declaration (as revised in 2013).

#### In-vitro cell culture

For fibroblast cultures, forearm punch biopsies were cut into small pieces, and the epidermal layer was removed. The small dermal biopsy parts were incubated in fibroblast culture medium (HAM’s F-12 Nutrient Mix (ThermoFisher) supplemented with 20% fetal bovine serum (FBS, Sigma), penicillin [100 UI/mL] and streptomycin [100 μg/mL]) (both ThermoFisher), in a humidified incubator at 37°C and 5% CO2. As soon as fibroblasts were growing from the biopsies, medium was changed to HAM’s F-12 nutrient mix with 10% FBS and fibroblasts were passaged every 3–4 days using trypsin-EDTA (Gibco).

### Method details

#### Fibroblast cultures

Dermal fibroblasts were obtained from forearm biopsies of patients and healthy controls. Primary dermal fibroblasts were cultured in culture medium (HAM F12) with 10% FBS, penicillin (100 UI/ml) and streptomycin (100 μg/mL), in a humidified incubator at 37°C and 5% CO2. Medium was changed every 3–4 days. Cells were split at 80% confluency using Trypsin-EDTA.

#### Enzyme activity assays

The activities of respiratory chain complex activities were measured at 37°C with a UVmc2 spectrophotometer on mitochondrial enriched fraction according to standard routine clinical protocols for CI (NADH:ubiquinone reductase, NUR), Complex II (Succinate:Ubiquinone reductase, SUR), Complex III (Ubiquinol:Cytochrome *c* Reductase, UCCR), Complex IV (Cytochrome *c* Oxidase, COX), Complex V (F1-ATPase) and Citrate synthase (CS). The protein content was determined with the bicinchoninic assay kit using bovine serum albumin as standard. Respiratory complex activities were normalized to CS as a mitochondrial content marker.^56^^,^^57^^,^^58^ Thirty-seven and 100 internal control samples were used in the experiments with muscle and fibroblasts, respectively.

#### Seahorse-based Oxygen consumption rate measurements

OCRs were measured using the Seahorse XFe96 Extracellular Flux analyzer (Agilent). Control and patient primary skin fibroblasts were seeded at 15 000 per well in cell culture medium supplemented with 10% FBS and 1% penicillin/streptomycin and grown overnight at 37°C with 5% CO2. One hour before measurement, culture medium was removed and replaced by Agilent Seahorse XF Base Medium complemented with 10 mM glucose, 1 mM sodium pyruvate, and 200 mM L-glutamine and incubated at 37°C, without CO2. Basal oxygen consumption was measured four times followed by three measurement cycles after each addition of 1 μM oligomycin A, carbonyl cyanide 4-(trifluoromethoxy) phenylhydrazone FCCP, and rotenone and antimycin A respectively. One measurement cycle consisted of 3 min of incubation and 3 min of measuring. OCR was normalized to CS activity. After completion of OCR measurements, the Seahorse assay medium was replaced by 0.33% Triton X-100, 10 mM Tris-HCl (pH 7.6), after which the plates were stored at −80°C. Before measurements, the plates underwent two thaw-freeze cycles and 3 mM acetyl-CoA, 1 mM 5,5′-dithiobis-2-nitrobenzoic acid (DTNB), and 10% Triton X-100 was added. Using a spectrophotometer. Background conversion of DTNB was measured at 412 nm and 37°C for 10 min at 1 min intervals. Hereafter, 10 mM of the CS substrate oxaloacetate was added to start the reaction. Subsequently, the ΔA412 nm was measured again for 10 min at 1 min intervals at 37°C. CS activity was calculated from the rate of DTNB conversion in the presence of substrate, from which the background DTNB conversion rate was subtracted, using an extinction coefficient of 0.0136 μmol/cm.

#### Imaging Flow Cytometry assays

Cells were plated in 6-well plates to reach 70–80% confluency. At the day of the assay, cells were incubated in the antibody mixture containing TMRM (30 nM), NAO (50 nM) and DRAQ5 (8000x diluted) at 37° in Hank's Balanced Salt Solution (HBSS) for 40 min. FCCP (3 μM) was added during the final 5 min. MitoSox (5 mM) staining was performed in a separate tube. After incubation, cells were washed once and incubated with TrypLE for 2 min. Cells were harvested using 1 mL 10% dialyzed FBS in PBS0, washed once, and immediately visualized using IFC. Since lipophilic cations like TMRM are extruded by Multi Drug Resistance transporters,^59^ TMRM fluorescence is not stable for prolonged periods.^60^ Therefore, we included a total of four samples per assay. All experiments were performed within 30 min after the staining procedure, and each assay was performed in the exact same order to keep the duration between staining and imaging similar.

#### Imaging Flow Cytometry features

To quantify mitochondrial morphology and function, six features were selected. TMRM intensity reflects the absolute mitochondrial membrane potential. The normalized membrane potential is calculated by subtracting TMRM intensity (Intensity_Ch03) in cells treated with FCCP (a potent mitochondrial uncoupler) from total TMRM intensity. FCCP response was calculated by dividing the intensity of TMRM of the non-treated sample by the intensity of TMRM in the treated sample. NAO intensity was used to indicate mitochondrial mass, correlating with the ability of cells to produce and maintain mtDNA. We calculated NAO intensity in the FCCP treated sample, to limit the effect of membrane potential on NAO intensity. For mitochondrial morphology, we used Form Factor, a measure reflective of mitochondrial fragmentation (decreased form factor) or compensatory branching (increased form factor). For the form factor, the spot mask on the TMRM staining was used (Bright, threshold 10, minimal area 0, maximal area 2). The median values derived from the Area_Spot_M03(10-0-2) and Perimeter Spot_M03(10-0-2) features were used for the calculation of the form factor: [(perimeterˆ2)/(4π·surface area)]. Mitochondrial swelling was calculated by measuring total Area of the TMRM staining and reflects the mitochondrial permeability transition pore functionality (mPTP). Mitochondrial ROS production was assessed by quantifying the cytoplasmic MitoSox intensity, to exclude intensity related to nonspecific binding of MitoSox to nucleic acids. For MitoSox intensity, a binary mask was created, that excluded the nuclear surface (DRAQ5 – Ch05 Morphology Mask), but included the cytoplasm (Ch01 Morphology) (Figure S3).

#### Molecular compounds used for assay validation

To validate the specificity of the membrane potential assay, we incubated cells with 10 ng/mL (25 μM) Rotenone 4 h prior to the assay. To stimulate mitochondrial biogenesis, we incubated cells with 10 mM Valproic acid for 4 days. To deplete mitochondrial mass, we incubated cells with 25 ng/mL Ethidiumbromide for 7 days. Ethididumbromide was also use to induce mitochondrial swelling. To induce mitochondrial fusion, we treated fibroblasts with 50 μM MDIVI for 16 h. To induce mitochondrial fragmentation, we incubated cells with 1.2 μM Staurosporine for 2 h prior to the assay. To induce mitochondrial ROS production, we stimulated cells with 1 μM antimycin A for 40 min.

### Quantification and statistical analysis

Data were collected with Amnis IFC MkII Imaging Flow Cytometer and data analysis was performed with IDEAS version 6.0 software. Magnification was set at 63X, and the lasers with excitation set at wavelengths 488 and 642 were used for all experiments, with power 10mW and 150mW respectively. The brightfield channel (430-480nM), channel 2 (505-560nM, for NAO), channel 3 (560-595nM,TMRM), and channel 5 (642-740nM, DRAQ5) were used. To create a compensation matrix, single stains of fibroblasts with either TMRM, NAO or DRAQ5 were used. MitoSox and DRAQ5 were not compensated for. Gating strategy can be found in Figure S3. Briefly, nucleated cells, cells in focus and single cells were gated. To create Figure 1, raw values were extracted from IDEAS as excel files and analyzed using using R and R-studio.

For Figure 2B, normalized percentage values were used, for which the median value of each sample was extracted from IDEAS. Subsequently, the median values of each patient were normalized against a healthy control sample analyzed within the same experiment. All graphs and UMAP plots were created using R-studio, except for the line chart of Figure 2A, which was created with GraphPad Prism version 6 for Windows. The following R packages were used for analysis: umap (0.2.10.0),^61^ dplyr (1.1.3),^62^ ggplot2 (3.4.3),^63^ ggfortify (0.4.16),^64^ gridExtra (2.3),^65^ corrplot (0.92),^66^ factoextra (1.0.7),^67^ ggradar (0.2).

For all statistical tests, the asterisks indicate the following: ∗*p* < 0.05, ∗∗*p* < 0.01, ∗∗∗*p* < 0.001, *∗∗∗∗p < 0.0001.* Details on statistical analysis can be found in Table S2.

## Supplemental information

Document S1. Figures S1–S3

Table S1. Overview of IFC results, related to Figures 2, 3, and 4 Overview of IFC results, clinical data and biochemical data for all healthy control and patient fibroblast lines. Blank columns indicate that values were not assessed (NA). The column “Seahorse” describes the results of mitochondrial stress test experiments using Agilent Seahorse equipment. All measurements were performed in a diagnostic setting at the Radboud MC, Nijmegen. Each patient was compared to at least three healthy controls. “Normal” indicates that no significant reduction in basal or maximal respiratory capacity was observed for patients compared to healthy controls. The “Oxphos activity measurement column” summarizes the results of the OXPHOS activity measurements, also performed in a diagnostic setting at the Radboud MC, Nijmegen. The results of these measurements are shown in the columns Y t/m AI. Absolute values were normalized to the lower reference range of healthy controls and depicted as percentage. The column “Age of onset” refers to the age where first symptoms were seen (shown in months). The columns K–V indicate the presence (1) or absence (0) of specific clinical features

Table S2. Details on statistical analysis, related to Figures 1, 2, 3, and 4; and Figure S2 Table S2A: Details on statistical analysis for Figure 1 Table S2B: Details on statistical analysis for Figure 2D Table S2C: Details on statistical analysis for Figure 3 Table S2D: Details on statistical analysis for Figures 4A, 4B, and 4C Table S2E: Details on statistical analysis for Figure S2
