# Supplementary material for: Imaging flow cytometry reveals divergent mitochondrial phenotypes in mitochondrial disease patients
Source: iScience. 2024 Nov 28;28(1):111496. doi: 10.1016/j.isci.2024.111496 (PMC11719857; doi:10.1016/j.isci.2024.111496)
Supplement: Document S1. Figures S1–S3 [file mmc1.pdf]

iScience, Volume *28*

Supplemental information

Imaging flow cytometry reveals divergent mitochondrial phenotypes in mitochondrial disease patients

Irena.J.J. Muffels, Richard Rodenburg, Hanneke L.D. Willemen, Désirée van Haaften-Visser, Hans Waterham, Niels Eijkelkamp, Sabine A. Fuchs, and Peter M. van Hasselt

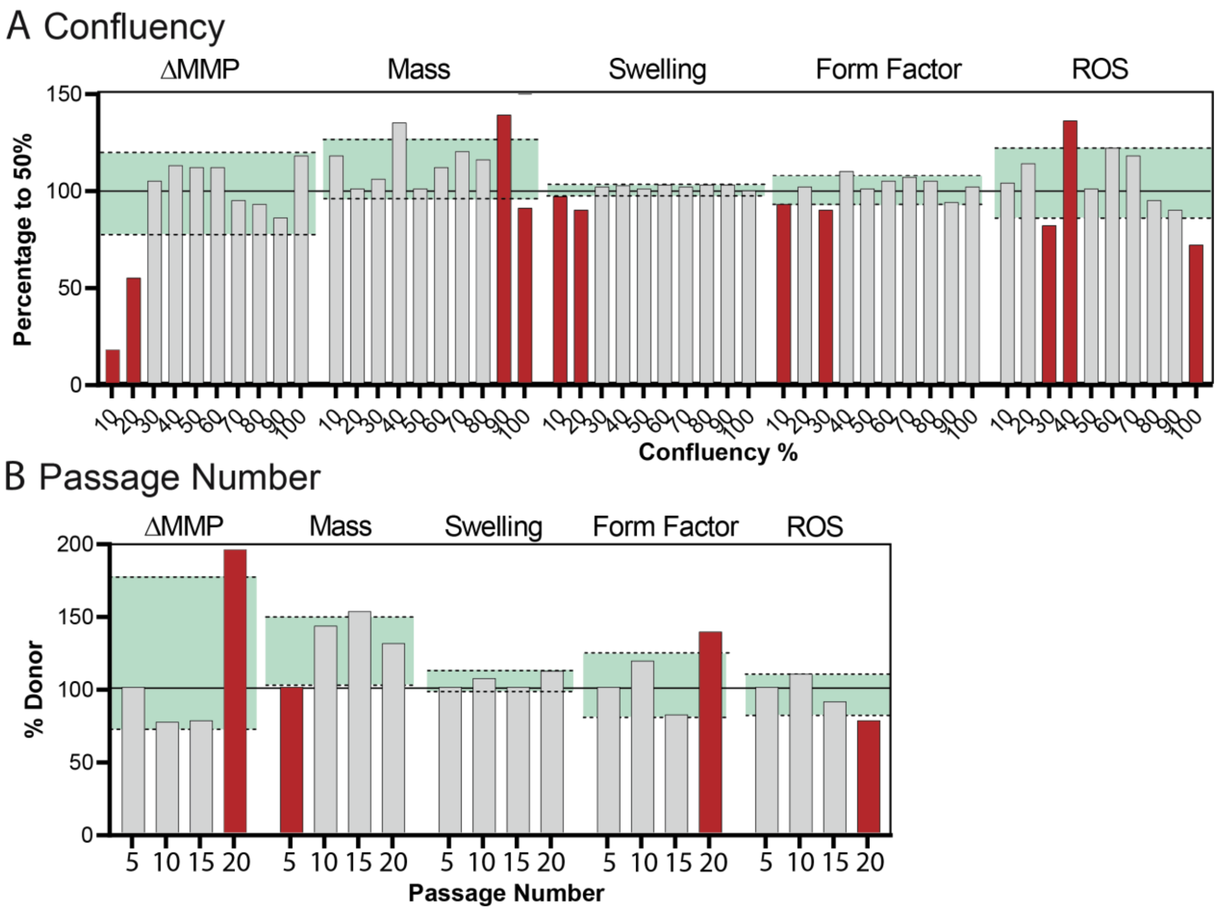

Figure S1: Effect of passage number and cellular density on IFC features.
(A) Bar chart showing the effect of fibroblast confluency right before harvesting on assay results. Each value was normalized to the feature value observed at 50% confluency taken along in the same run and converted to percentages. The green planes indicate the IQR25-IQR75 range. The red colored bars indicate that the values fall outside (>5%) the IQR25-IQR75 range.
(B) Bar chart showing the effect of fibroblast passage number on assay results. Each value was normalized to passage number 5, taken along in the same run and converted to percentages. The green planes indicate the IQR25-IQR75 range. The red colored bars indicate that the values fall outside (>5%) the IQR25-IQR75 range.

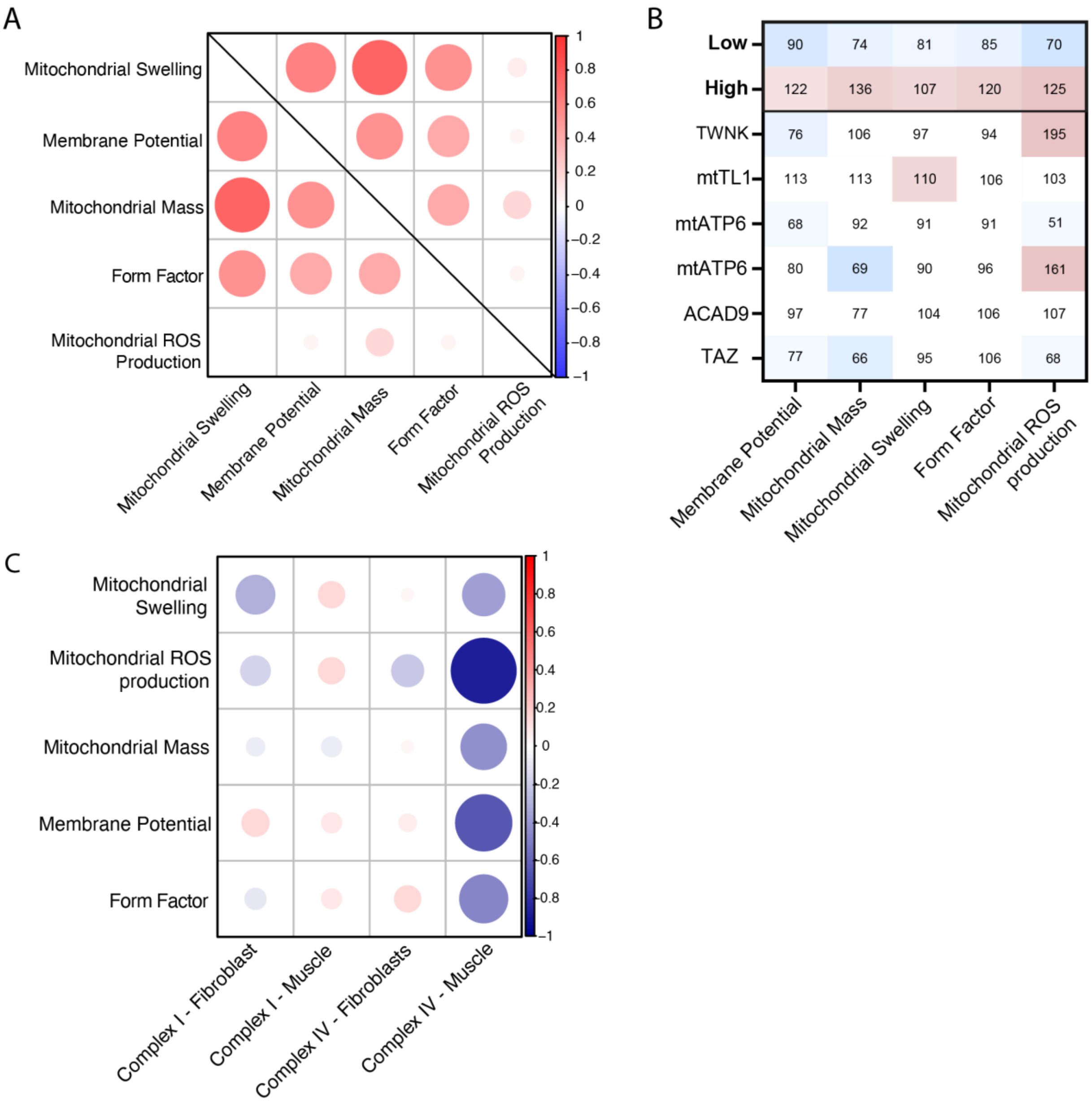

Figure S2: Comparison of IFC assays with other diagnostic tools

(A) Correlation plot showing correlation between IFC assays. Correlation was calculated using Spearman statistics. None of the rho correlation statistics was >0.7. However, correlation between mitochondrial swelling and membrane potential was high, with rho 0.64 and p=0.0001.

(B) Heat map showing assay results in mitochondrial disease patients where seahorse respirometry was performed. All patients had normal basal, spare- and maximal respiratory capacity (N=6). All values are shown as percentage normalized against the healthy control taken along in the same run. The top two rows indicate the lowest- and highest reference values found in six healthy controls. The color coding refers to the severity of the aberrancies. White boxes indicate that the value falls within the range (lowest-highest value) of healthy controls. Colored boxes indicate that the value falls outside the range observed in healthy controls.

(C) Correlation plot showing the correlation between IFC features and OXPHOS complex activities measured in fibroblast or muscle. All patients where OXPHOS complex activity was measured were included for analysis (N=6 for muscle, N=13 for fibroblasts). Correlation was calculated using Spearman. The correlation between ROS production and complex IV activity in muscle was statistically significant (p=0.03).

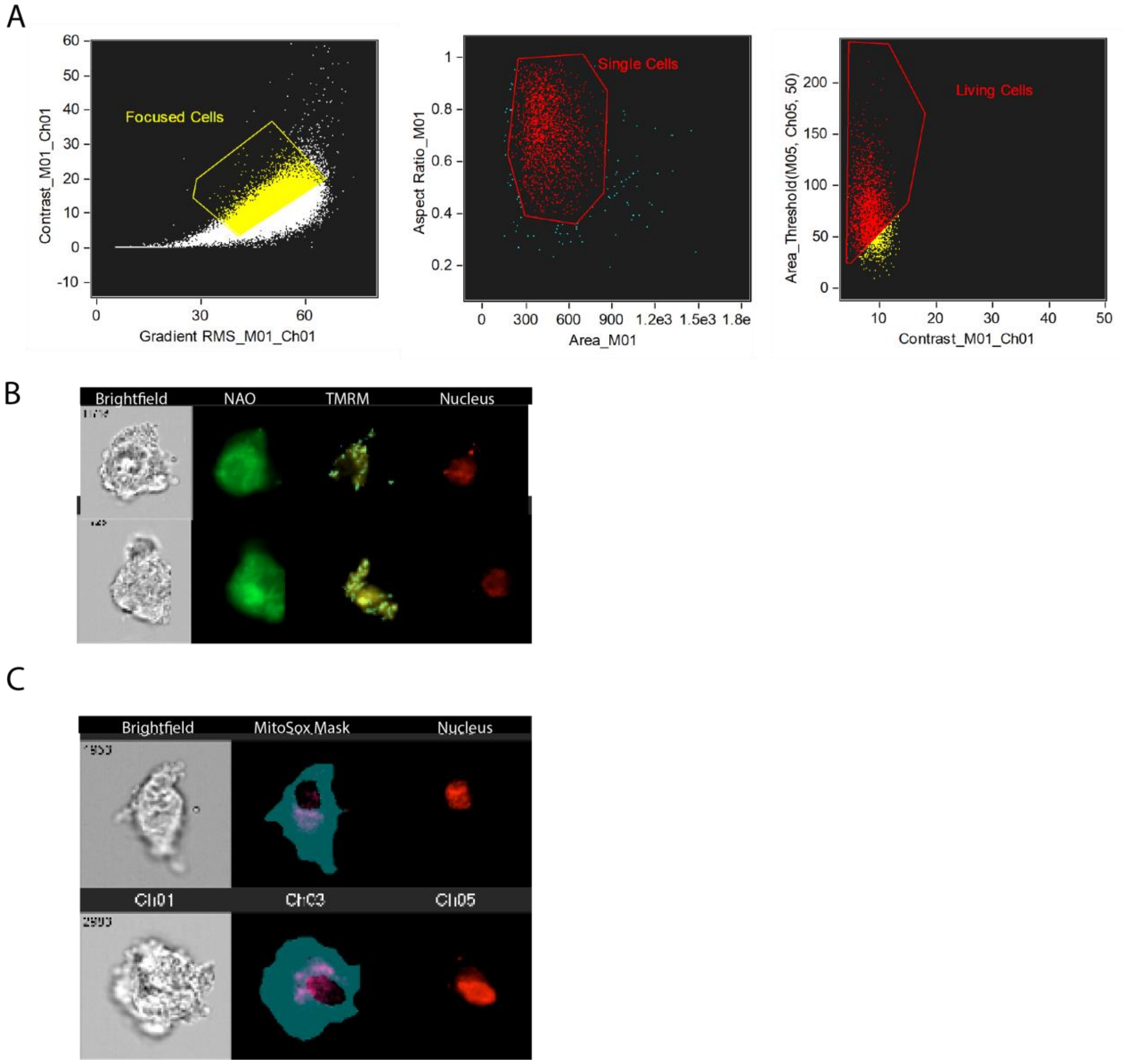

Figure S3: Gating Strategy

(A) Primary gating applied on all samples.

(B) Showing TMRM mask used to calculate the form factor. The Spot mask was used to remove the background of the TMRM staining. The threshold was set to 10, the minimum size to 0 pixels and maximum size to 2 pixels.

(C) Showing the mask used to quantify mitochondrial ROS production. A mask excluding the nuclear pixels was created by combining the morphology mask of the brightfield image and excluding the morphology mask of the nuclear staining.
